# Cohesin’s DNA Exit Gate Is Distinct from Its Entrance Gate and Is Regulated by Acetylation

**DOI:** 10.1016/j.cell.2012.07.028

**Published:** 2012-08-31

**Authors:** Kok-Lung Chan, Maurici B. Roig, Bin Hu, Frédéric Beckouët, Jean Metson, Kim Nasmyth

**Affiliations:** 1 University of Oxford, Department of Biochemistry, South Parks Road, Oxford OX1 3QU, UK

## Abstract

Sister chromatid cohesion is mediated by entrapment of sister DNAs by a tripartite ring composed of cohesin’s Smc1, Smc3, and α-kleisin subunits. Cohesion requires acetylation of Smc3 by Eco1, whose role is to counteract an inhibitory (antiestablishment) activity associated with cohesin’s Wapl subunit. We show that mutations abrogating antiestablishment activity also reduce turnover of cohesin on pericentric chromatin. Our results reveal a “releasing” activity inherent to cohesin complexes transiently associated with Wapl that catalyzes their dissociation from chromosomes. Fusion of Smc3’s nucleotide binding domain to α-kleisin’s N-terminal domain also reduces cohesin turnover within pericentric chromatin and permits establishment of Wapl-resistant cohesion in the absence of Eco1. We suggest that releasing activity opens the Smc3/α-kleisin interface, creating a DNA exit gate distinct from its proposed entry gate at the Smc1/3 interface. According to this notion, the function of Smc3 acetylation is to block its dissociation from α-kleisin. The functional implications of regulated ring opening are discussed.

## Graphical abstract

**Graphical Abstract:**

**Figure:**
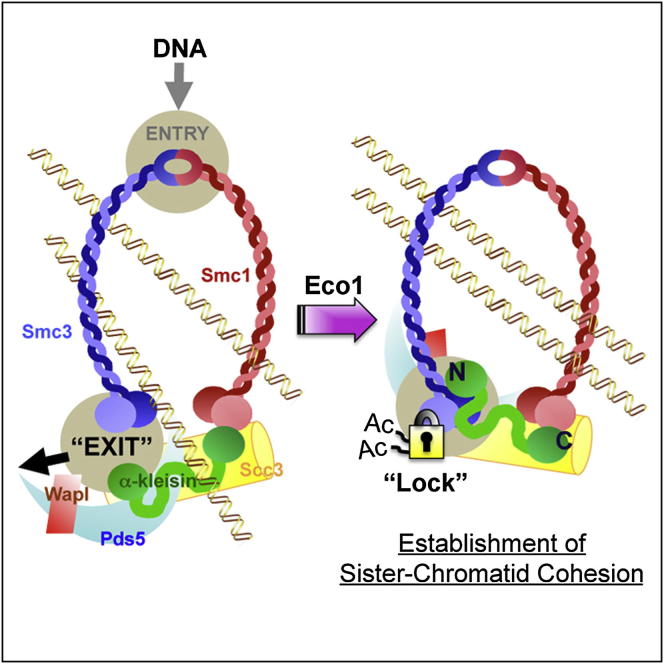

**Highlights:**

► Yeast cohesin releases DNA through a releasing activity associated with its subunits ► DNA escapes from cohesin via the opening Smc3/kleisin interface ► Eco1-mediated Smc3 acetylation blocks releasing activity ► Cohesin ring has separate gates for DNA entry and exit

## Teaser

Cohesin rings entrap and then release sister chromatids using conformational changes in distinct ring subunits to create the entrance and exit sites.

## Introduction

The sister chromatid cohesion essential for orderly chromosome segregation during mitosis and meiosis is mediated by a multisubunit complex called cohesin (Guacci et al., 1997; Michaelis et al., 1997), whose Smc1 and Smc3 subunits are rod-shaped proteins with an ABC-like nucleotide binding domain (NBD) at one end and a dimerization domain at the other (Haering et al., 2002). Interactions between the latter generate V-shaped Smc1/3 heterodimers with a “hinge” at the base of the V and NBDs at its vertices. Association of Smc1 and Smc3 NBDs with the C- and N-terminal domains of cohesin’s α-kleisin subunit, respectively, generate large tripartite rings within which sister DNAs can be entrapped (Haering et al., 2008). This process takes place during DNA replication and is accompanied by and depends on acetylation of Smc3’s NBD by the Eco1 acetyltransferase (Nasmyth, 2011).

Cohesin associates with unreplicated as well as replicated chromatin fibers. In mammalian cells, a major increase in the residence time of a fraction of the cohesin pool accompanies DNA replication (Gerlich et al., 2006). It is presumed that this stable fraction is engaged in holding sister DNAs together. De novo association, known as cohesin loading, depends on hydrolysis of ATP bound to Smc1 and Smc3 NBDs (Arumugam et al., 2003; Weitzer et al., 2003) on a fourth subunit called Scc3 (Hu et al., 2011), which binds to the central domain of α-kleisin (Haering et al., 2002), and on a separate complex called kollerin (Nasmyth, 2011), containing the Scc2 and Scc4 proteins (Ciosk et al., 2000). Available evidence suggests that this process involves transient dissociation of the Smc1/3 hinge dimerization interface, which acts as a DNA entry gate (Gruber et al., 2006).

Dissociation from chromosomes takes place via two pathways. The cohesin associated with mitotic chromosomes that permits their biorientation on mitotic spindles is removed after congression through cleavage of its α-kleisin subunit by separase (Uhlmann et al., 1999; Uhlmann et al., 2000). This opens the ring irreversibly, permitting escape of DNAs previously trapped inside (Ivanov and Nasmyth, 2007). A different process called the prophase pathway causes dissociation of most cohesin associated with chromosome arms as cells enter mitosis via a separase independent mechanism (Losada et al., 1998; Waizenegger et al., 2000). If cohesin associates with chromatin by encircling DNA, then release during prophase must involve ring opening due to dissociation of one of its three interfaces. Neither the existence nor identity of this “exit gate” is known.

The prophase pathway depends on Wapl (Gandhi et al., 2006; Kueng et al., 2006), a protein that binds to Pds5, a large HEAT-repeat-containing protein that, like Scc3, is recruited to the ring by binding α-kleisin (Hartman et al., 2000; Panizza et al., 2000; Shintomi and Hirano, 2009). Although cohesin’s dissociation from chromatin mediated by Wapl is greatly increased as cells enter mitosis, it also occurs throughout the cell cycle (Gerlich et al., 2006). Thus, the fraction of cohesin associated with chromatin as well as its residence time is determined by the relative activities of kollerin, which catalyzes association, and a releasing activity inherent to cohesin complexes associated with Wapl, which catalyzes dissociation. Though yeast has a Wapl ortholog, it lacks a discernable prophase pathway and as a result most if not all cohesin is cleaved by separase.

The Eco1 acetyltransferase is unnecessary for cohesin’s association with chromatin but essential for generating cohesion during DNA replication (Skibbens et al., 1999; Tóth et al., 1999). A key insight into its role stemmed from the finding that the lethality of *eco1* null alleles can be suppressed by mutations in a number of cohesin subunits. Thus, deletion of *pds5*, which is not an essential gene in *Schizosaccharomyces pombe*, suppresses lethality caused by deletion of *eso1*, *S. pombe*’s Eco1 ortholog (Tanaka et al., 2001). This unexpected finding suggested that in addition to promoting cohesion, Pds5 has a negative function that interferes with cohesion and that a key function of Eso1 is to counteract this. A similar phenomenon has since been analyzed in *Saccharomyces cerevisiae* where highly specific *smc3*, *pds5*, and *scc3* missense mutations or *wpl1* null alleles have the same property (Rolef Ben-Shahar et al., 2008; Rowland et al., 2009; Sutani et al., 2009; Unal et al., 2008; Zhang et al., 2008a). The *smc3* suppressors include K113, one of two amino acids acetylated by Eco1, as well as neighboring residues within Smc3’s NBD, whereas clusters of *pds5* and *scc3* suppressor mutations define specific domains within the N- and C-terminal halves of these two cohesin subunits (Rowland et al., 2009). It has therefore been suggested that a key function of Smc3 acetylation by Eco1 is to neutralize an “antiestablishment” activity associated with parts of the cohesin complex identified by *eco1* suppressor mutations, an activity that interferes with either creation or maintenance of cohesion (Rowland et al., 2009). The finding that sororin (Rankin et al., 2005), whose recruitment to cohesin rings in animal cells accompanies Smc3 acetylation, blocks Wapl’s ability to bind Pds5 is further evidence that neutralizing an activity dependent upon Wapl is a key function of acetylation (Lafont et al., 2010; Nishiyama et al., 2010).

Because Wapl in mammalian cells promotes cohesin’s dissociation from chromosome arms during prophase, it has been suggested that “antiestablishment” is caused by an activity inherent to cohesin complexes that promotes their dissociation from chromatin fibers (Rolef Ben-Shahar et al., 2008; Rowland et al., 2009; Sutani et al., 2009; Unal et al., 2008; Zhang et al., 2008a). According to this notion, the role of acetylation is to neutralize a releasing activity that promotes escape of DNAs from tripartite cohesin rings by opening the Smc1/Smc3, Smc1/α-kleisin, or Smc3/α-kleisin interface. However, this notion is not without its problems. Yeast does not possess a prophase pathway. Moreover, its Wapl ortholog has not so far been implicated in cohesin turnover. Indeed, Wapl inactivation actually decreases, not increases, the amount of cohesin on yeast chromosomes (Rowland et al., 2009; Sutani et al., 2009). These difficulties have therefore led to an alternative explanation for antiestablishment, namely that it interferes with the process by which sister DNAs are initially entrapped by cohesin rings during DNA replication (Rowland et al., 2009; Sutani et al., 2009).

The notion that Eco1’s primary function is to block cohesin’s release from chromatin even in yeast makes a number of predictions. First, Wapl should be capable of destroying cohesion long after its establishment. There is evidence that this occurs in *Xenopus* extracts (Shintomi and Hirano, 2009) but no evidence hitherto in cellular systems, including yeast. Second, all *eco1* suppressor mutations that define antiestablishment should be defective in releasing cohesin from yeast chromosomes. Third and most crucial, if releasing activity functions by promoting escape of DNAs from within cohesin rings, then these must have a defined exit gate that if sealed should block release and thereby mimic the effects of Eco1. This paper describes experiments that address all three predictions. Our finding that fusion of Smc3’s NBD to the N-terminal domain of α-kleisin permits establishment of cohesion in the absence of Eco1 confirms that DNAs are indeed entrapped by cohesin rings and implies that releasing activity functions by opening an exit gate at the Smc3/kleisin interface. This key observation suggests that cohesin has separate DNA entry and exit gates.

## Results

### Wapl Destroys Cohesion after DNA Replication

To address whether antiestablishment activity prevents cohesin from embracing sister DNAs in the first place or whether it destroys such structures after they have been produced, we created an *eco1Δ* yeast strain in which Wapl is expressed from the *GAL* promoter (*GAL-WPL1*). These cells proliferate when grown in the absence of galactose but cease to do so upon its addition, which induces Wapl expression. They harbored a 7.5 kb circular minichromosome whose sister DNA cohesion (cohesion-mediated dimers) can be measured by physical means using differential sedimentation velocity and gel electrophoresis (Farcas et al., 2011).

**Figure 1:**
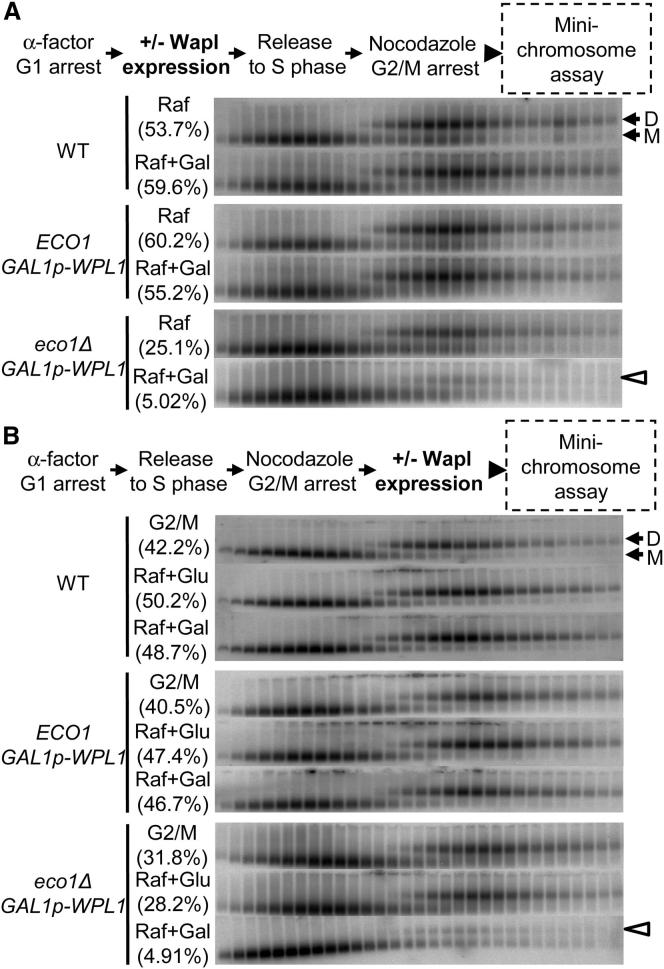
Wapl Destroys Cohesion after DNA Replication Minichromosome dimers and monomers were separated by sucrose gradient sedimentation and gel electrophoresis and detected by Southern blotting. (A) Strains K17615, K18942, and K18943 were incubated with α-factor for 1.5 hr in YEP raffinose media and cultures split; 2% galactose was added to one, which induced Wapl. Cells were subsequently incubated in media lacking pheromone but containing 10 μg/ml nocodazole and harvested after 90 min. (B) As for (A), except Wapl expression was induced by adding galactose 90 min after release from pheromone. (“D” and “M” denote dimeric and monomeric minichromosomes, respectively; arrowheads indicate loss of minichromosomes dimers; Brackets denote percentages of DNAs in dimeric fractions).

Wild-type (*ECO1 WPL1*), *ECO1 GAL-WPL1*, or *eco1Δ GAL-WPL1* cells growing in noninducing raffinose medium were arrested in G1 by incubation in the presence of pheromone and then released into medium containing galactose and nocodazole. Under these conditions, cells undergo replication and arrest in a mitotic state. Induction of Wapl from the *GAL* promoter had little or no adverse effect on cohesion produced by *ECO1* cells and prevented its creation in *eco1Δ GAL-WPL1* cells (Figure 1 A). The experiment was repeated with an important variation, namely galactose was only added after cells had undergone DNA replication. Induction of Wapl in this manner had no effect on cohesion established by wild-type or *ECO1 GAL-WPL1* cells but greatly reduced minichromosome cohesion in *eco1Δ GAL-WPL1* cells (Figure 1B). This result demonstrates that an activity dependent on Wapl does not merely (if at all) prevent creation of cohesion; it actually destroys cohesion that has already been established. Importantly, Wapl cannot do this if Smc3 has been acetylated by Eco1. Antiestablishment is therefore capable of acting long after DNA replication. Importantly, denaturation of dimer fractions before gel electrophoresis demonstrated that they were largely composed of monomeric DNAs held together by cohesin and not DNA-DNA catenation (data not shown).

### Wapl Associates with Cohesin in a Substoichiometric Manner

To understand better how Wapl destroys cohesion, we addressed the nature of its association with chromosomal cohesin. To do this, we created strains in which cohesin subunits including Wapl were tagged with GFP or RFP. Proliferation of haploids (or homozygous diploids) with tagged cohesin subunits was in each case indistinguishable from wild-type, implying that the fusion proteins were functional.

**Figure 2:**
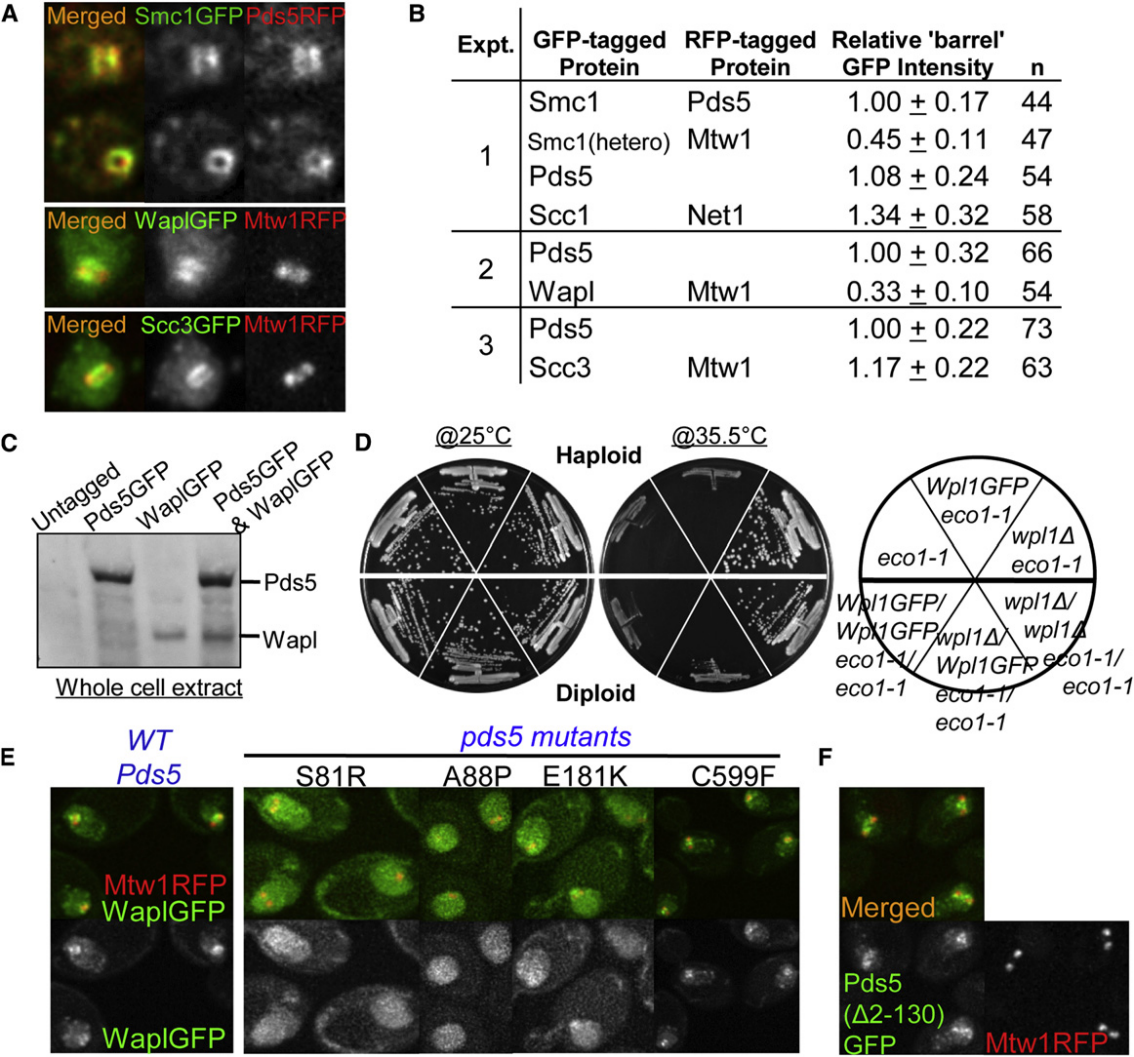
Wapl Is Substoichiometric (A) Live images of GFP-tagged Smc1, Pds5 (K17792), Wapl (K18804), and Scc3 (K18110) forming pericentric barrels in wild-type G2/M diploids. (B) Relative GFP intensities of cohesin subunits at pericentric regions in living wild-type G2/M diploids (K17792, K19367, K19003, K18785, K18396, and K18110). Fluorescence was quantified on the same slides containing yeast strains expressing different cohesin subunits tagged with GFP. All strains except Smc1(hetero) were homozygous. The identity of strains was determined by using different RFP-tagged proteins. Three sets of quantitation experiments were performed with different combinations of yeast strains. Seventeen Z stacking images were acquired with 0.2 μm intervals. Data are represented as mean of GFP intensity ± standard deviation (SD). In *eco1-1* diploids, the relative mean GFP intensities ± SD of Scc1 (K19004) and Wapl (K18420) to that of Pds5 (K19005) (1.00 ± 0.28; n = 107) were 1.12 ± 0.28; n = 103 and 0.25 ± 0.08; n = 106, respectively. n = number of cells examined. (C) SDS-PAGE showing relative protein levels in strains containing either Wapl or Pds5, or both Wapl and Pds5 tagged with GFP (K16574, K17180, K18714, and K18516). (D) Heterozygous Wapl-GFP over a deletion is sufficient to cause lethality in *eco1-1* diploids at restrictive temperature (haploid: K18335, K18417, K19001; diploid: K18420, K19040, K19039). (E) Suppressor mutations in the N-terminal region of Pds5 abolish pericentric Wapl recruitment. Live-cell imaging shows localization of Wapl-GFP localization in wild-type (K18396) or *pds5* mutants S81R (K19125), A88P (K19200), E181K (K19192), and C599F (K19199). (F) N-terminal region of Pds5 is not required for its pericentric localization (K19105). See also Figure S1 for Scc1 dependence of the pericentric localization of Pds5, Wapl, and Scc3.

**Figure S1:**
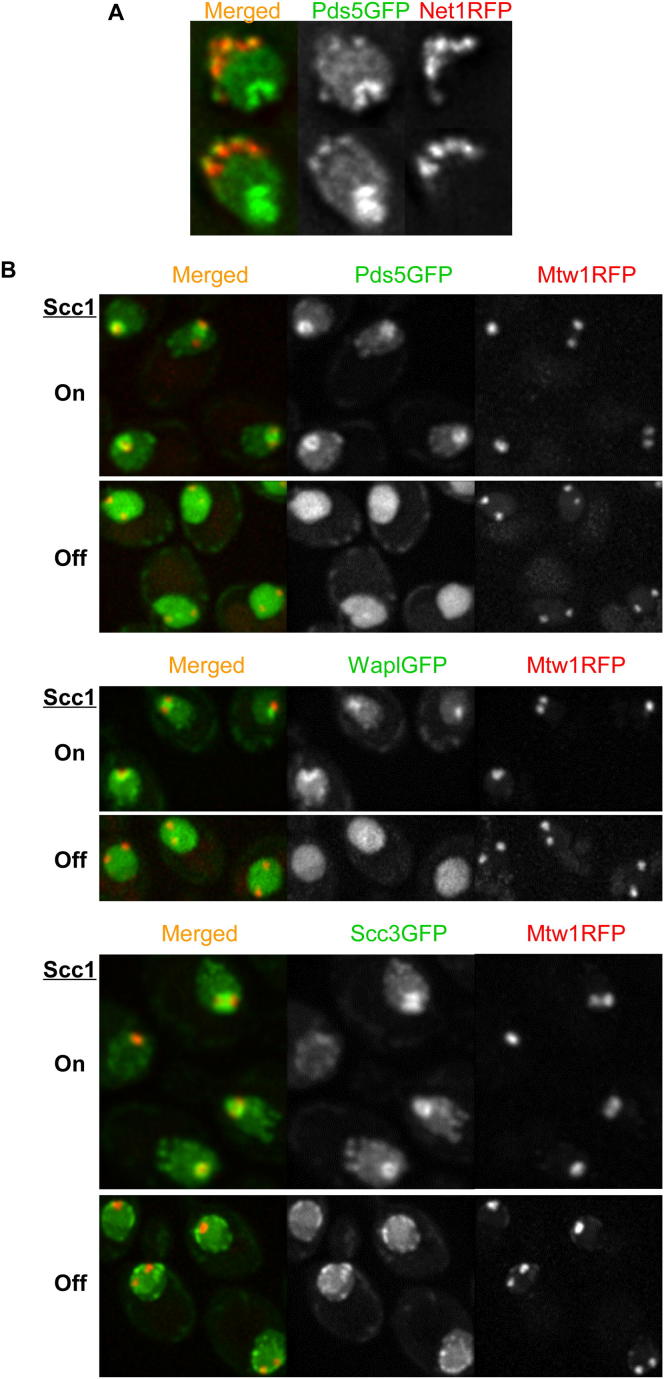
Subnuclear Localization of Cohesin Subunits Depends on α-Kleisin, Related to Figure 2 (A) Live-cell imaging showing the localization of Pds5-GFP at ribosomal DNA regions marked by Net1RFP (K18329). (B) The subnuclear localization of Wapl (K18848), Pds5 (K17373) and Scc3 (K17749) is dependent on Scc1. Endogenous Scc1 is under the control of *GAL* inducible promoter. Live-cell imaging was performed before and after switching from galactose to glucose for 1.5 hr at 25°C.

Resynthesis of its α-kleisin subunit Scc1 in late G1 induces loading of cohesin in the vicinity of centromeres and to a lesser extent along chromosome arms. Pericentric cohesin subsequently forms barrel-shaped structures around mitotic spindles following DNA replication and sister kinetochore bi-orientation (Yeh et al., 2008), which was visualized by using a RFP-tagged kinetochore protein (Mtw1-RFP). Wapl as well as Scc3 and Pds5 formed pericentric barrels similar to those formed by core tripartite ring subunits (Figure 2A). Live-cell imaging also detected cohesin subunits at ribosomal DNAs (Figure S1A available online). Importantly, enrichment of Wapl, Pds5, and Scc3 within pericentric chromatin was abolished upon depletion of cohesin’s α-kleisin subunit (Figure S1B).

Stoichiometry was compared by quantitating (on the same slide) the pericentric fluorescence of cells expressing different cohesin subunits tagged with GFP. The identity of each cell was determined by using RFP markers (Figure 2B). This method was validated by showing that fluorescence associated with the barrels of diploids heterozygous for the Smc1-GFP allele was about half that of homozygotes. Fluorescence of Pds5-GFP, Scc3-GFP, and Smc1-GFP barrels was very similar, whereas that of Scc1-GFP was slightly greater. The values are consistent with a 1:1:1:1 stoichiometry, which is inconsistent with the suggestion that two tripartite rings bind a single Scc3 subunit (Zhang et al., 2008b). Surprisingly, fluorescence of Wapl-GFP barrels was one third that of Pds5, which is thought to be its immediate partner (see below).

To address whether the reduced levels of Wapl are a consequence of Smc3 acetylation, we compared Pds5-GFP and Wapl-GFP fluorescence associated with pericentric regions in ts *eco1-1* mutants incubated at the restrictive temperature. Due to the lack of cohesion under these conditions, kinetochores and associated pericentric chromatin disjoin (though frequently with sisters at the same pole) and cells accumulate with two distinct foci of fluorescence associated with each spindle pole (see below). Pericentric Pds5-GFP fluorescence associated with poles was very similar to that of Scc1-GFP. Importantly, both were four times greater than Wapl-GFP (Figure 2B). Quantitative western blotting confirmed that the total amount Wapl-GFP protein within cells is about four times lower than that of Pds5-GFP (Figure 2C), a result also obtained with myc-tagged proteins (data not shown). Despite Wapl’s low abundance and its substoichiometric association with pericentric cohesin, GFP-tagged or myc-tagged Wapl, like wild-type, caused lethality in *eco1-1* strains grown at the restrictive temperature (35.5°C) (Figure 2D and data not shown). Remarkably, Wapl-GFP caused lethality in *eco1-1* diploid cells even when heterozygous over *wpl1Δ* deletion (Figure 2D). These results suggest that Wapl manages to destroy all cohesion when Smc3 acetylation is compromised despite being associated at any one time with less than one-third of the chromosomal cohesin population.

### Wapl’s Recruitment Depends on Pds5’s N-Terminal Domain

Two pieces of evidence suggest that Wapl’s recruitment to pericentric cohesin depends on its association with Pds5. First, Wapl binds Pds5 in vitro (Kueng et al., 2006; Rowland et al., 2009; Shintomi and Hirano, 2009), and second, mutations within Scc1 defective in recruiting Pds5 also abrogate Wapl’s recruitment (unpublished observations). Reasoning that Pds5 mutations defective in binding Wapl should share with *wpl1Δ* the ability to suppress *eco1*, we analyzed the distribution of Wapl-GFP in a variety of Pds5 mutations known to suppress *eco1-1* (Rowland et al., 2009). Pds5S81R and A88P abolished association of Wapl-GFP with pericentric DNAs, whereas C599F had little effect (Figure 2E), suggesting that Wapl is recruited by Pds5’s N-terminal domain. Importantly, the effect on Wapl recruitment of Pds5S81R and A88P cannot be attributed to a corresponding lack of Pds5 recruitment because a version of Pds5 lacking its N-terminal domain (Δ2-130) does not affect its pericentric recruitment (Figure 2F). Consistent with a role in recruiting Wapl, this domain is not essential for yeast cell proliferation despite being the most conserved part of Pds5.

### Turnover of Pds5 but Not Wapl Is Regulated by Eco1

**Figure 3:**
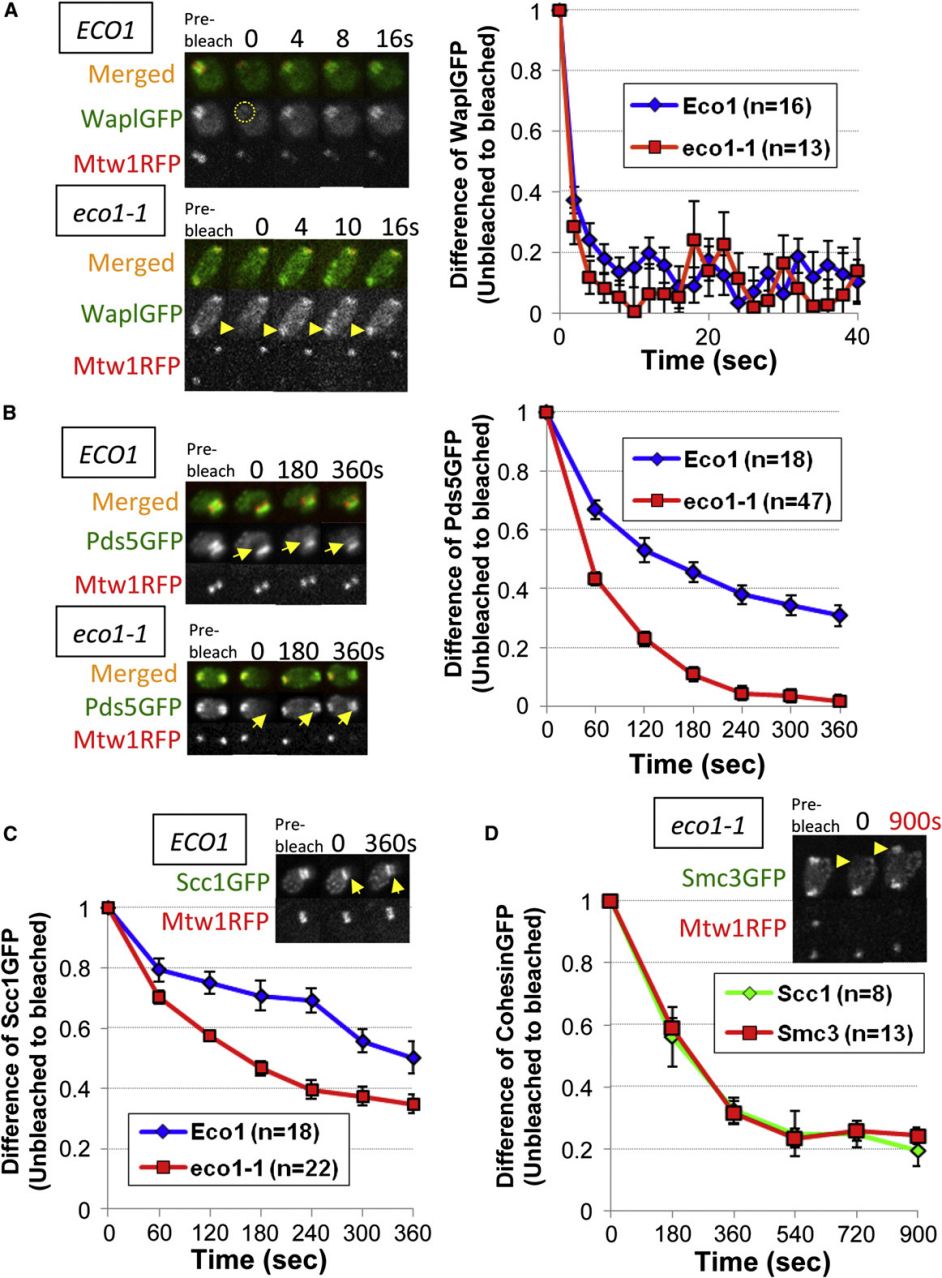
Turnover of Pds5 but Not Wapl Is Regulated by Eco1 (A) The entire Wapl-GFP barrel region was photobleached (indicated by a dotted circle) in wild-type tetraploid cells (K18804) and single-stacking images acquired every 2 s. In *eco1-1* diploids (K18420), one of the two sister-centromere clusters, was photobleached (indicated by arrows) after preincubation at nonpermissive temperature for 90 min. (B) One half of Pds5-GFP barrels in tetraploids (K18407) or one of two foci in *eco1-1* diploids (K18419) were photobleached (indicated by arrows) and five Z stacking images with 0.4 μm intervals undertaken every 60 s for 360 s. (C) Dynamics of Scc1 in wild-type tetraploids (K18246) and *eco1-1* diploids (K18402). Photobleaching and imaging was done as (B). (D) Cohesin dynamics in *eco1-1* cells over an extended period. Photobleaching and imaging was done as in (B), but imaging was done after photobleaching for 900 s in Smc3-GFP (K18454) and Scc1-GFP (K18402) *eco1-1* diploids. Data are represented as mean difference of normalized fluorescence intensity between bleached and unbleached regions ± SEM. n = the number of cells examined. Sister-centromere clusters are marked by Mtw1RFP. See also Figure S2 for normalized fluorescence intensity of unbleached and bleached regions.

**Figure S2:**
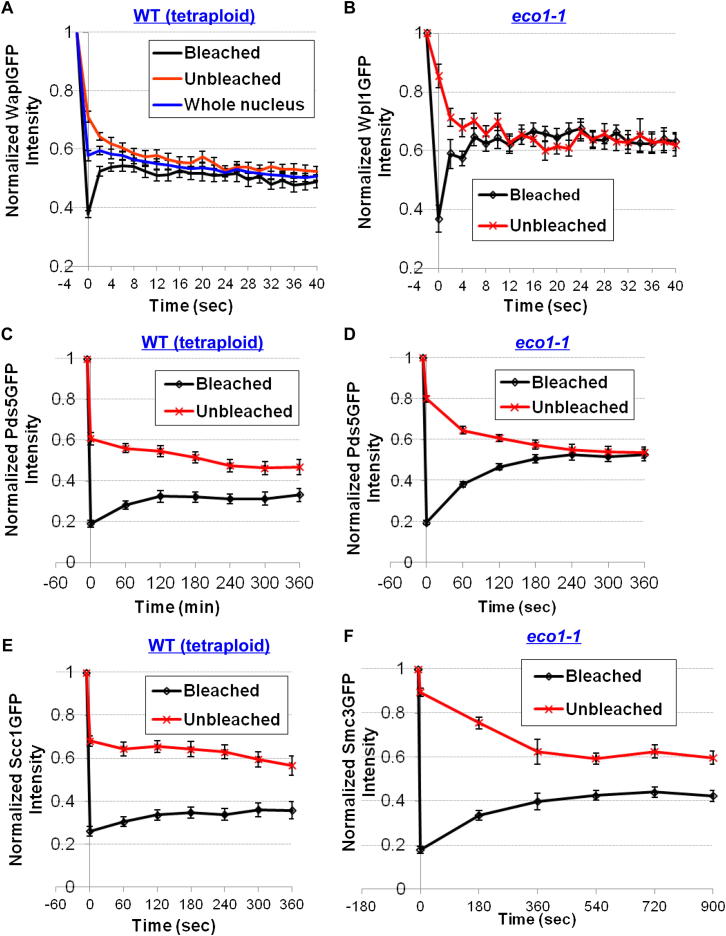
Dynamics of Cohesin and Its Regulatory Subunits in Wild-Type Tetraploids and *eco1-1* Diploids, Related to Figure 3 (A) Normalized fluorescence intensity of the unbleached, bleached regions and of the whole nuclei in wild-type tetraploids expressing WaplGFP (K18804). (B) Normalized fluorescence intensity of the unbleached and bleached centromere clusters in *eco1-1* diploids expressing WaplGFP (K18420). (C) Normalized fluorescence intensity of the unbleached and bleached halves of barrel in wild-type tetraploids expressing Pds5GFP (K18407). (D) Same as (B), except normalized Pds5GFP intensity is shown (K18419). (E) Same as (C), except normalized Scc1GFP intensity is shown (K18246). (F) Same as (B), except normalized Smc3GFP intensity is shown over an extended period after photobleaching (K18454). Data are represented as mean normalized fluorescence intensity (to prebleaching regions) ± SEM. n = the number of cells examined.

One explanation for Wapl’s ability to act substoichiometrically is that it acts catalytically, modifying cohesin through transient interactions. To test this, we used fluorescence recovery (FRAP) or persistence (iFRAP) after photobleaching to compare the dynamics of Wapl’s association with chromosomes with those of other cohesin subunits. We performed some of these studies with tetraploid cells that have larger nuclei and higher GFP intensities. Strikingly, fluorescence associated with Wapl-GFP barrels reappeared rapidly after photobleaching. Using the unbleached part of the nucleus as a reference, the difference in relative fluorescence between the bleached barrels and the unbleached chromatin decayed with a t_1/2_ < 2 s (Figure 3A; Figure S2A).

To address whether Wapl’s rapid turnover is due to acetylation of Smc3, we performed iFRAP in diploid *eco1-1* cells that had undergone DNA replication at the restrictive temperature and in which barrels are replaced by symmetrical pericentric foci associated with spindle poles. The distribution of these foci enabled us to photobleach one and to use its unbleached partner as a reference. Remarkably, fluorescence associated with bleached and unbleached foci converged within seconds (Figure S2B), again implying a t_1/2_ < 2 s (Figure 3A). This rapid exchange is not due to relocation of sister-centromere clusters because there is little recovery of Mtw1RFP. Wapl therefore exchanges rapidly between different pericentric cohesin complexes in a manner largely independent of Eco1, and this property may enable it to regulate a several fold larger cohesin population.

We next addressed whether Wapl’s partner Pds5 has a similarly dynamic association. The size and intensity of GFP fluorescence associated with pericentric chromatin in tetraploid cells enabled us to photobleach specifically one-half of their barrels and to acquire multi-Z-stacking images covering the entire 1.6 μm depth of nuclei (Figure 3B). The difference in fluorescence between bleached and unbleached regions initially decayed with a t_1/2_ of ∼60 s, implying rapid turnover of a large fraction of pericentric Pds5-GFP. However, the fluorescence of unbleached regions remained substantially greater than their bleached counterparts at the end of the imaging period (Figure S2C), implying a second population of molecules that turns over much more slowly. Unlike Wpl1, at least one-third of Pds5 is quite stably bound to pericentric chromatin in G2/M cells. Strikingly, the entire Pds5 population turns over with a t_1/2_ of ∼60 s in *eco1-1* mutants incubated at the restrictive temperature (Figure 3B; Figure S2D). We draw two conclusions from these findings. First, the extremely rapid Wapl turnover cannot be driven by Pds5 turnover. Second, acetylation, presumably of Smc3 NBDs by Eco1, creates a sizeable pool of pericentric cohesin in which Pds5 turns over only very slowly. It seems likely that this corresponds to the pool engaged in stably holding sister chromatids together.

### Turnover of Tripartite Ring Subunits

We next compared the chromosome dynamics of Wapl and Pds5 with that of core subunits. Previous studies using haploids or diploids (and a less accurate imaging system) had failed to detect turnover of tripartite ring constituents within the pericentric barrels of G2/M phase cells (Mishra et al., 2010; Yeh et al., 2008). However, the improved images from tetraploids revealed that approximately half of Scc1-GFP turns over with a t_1/2_ of ∼120 s (Figure 3C; Figure S2E). Fluorescence recovery is not simply due to barrel rotation because it also occurs after photobleaching the entire structure (data not shown). This dynamic population is presumably not engaged in cohesion because complexes holding sisters together do not exchange with those that load after S phase (Haering et al., 2004). The mobile fraction was even greater in *eco1-1* mutants (Figure 3C and 3D; Figure S2F). It should however be noted that one-fifth of pericentric cohesin fails to turn over even in *eco1-1* mutants (Figure 3D).

### Pericentric Cohesin Turnover Depends on Wpl1

**Figure 4:**
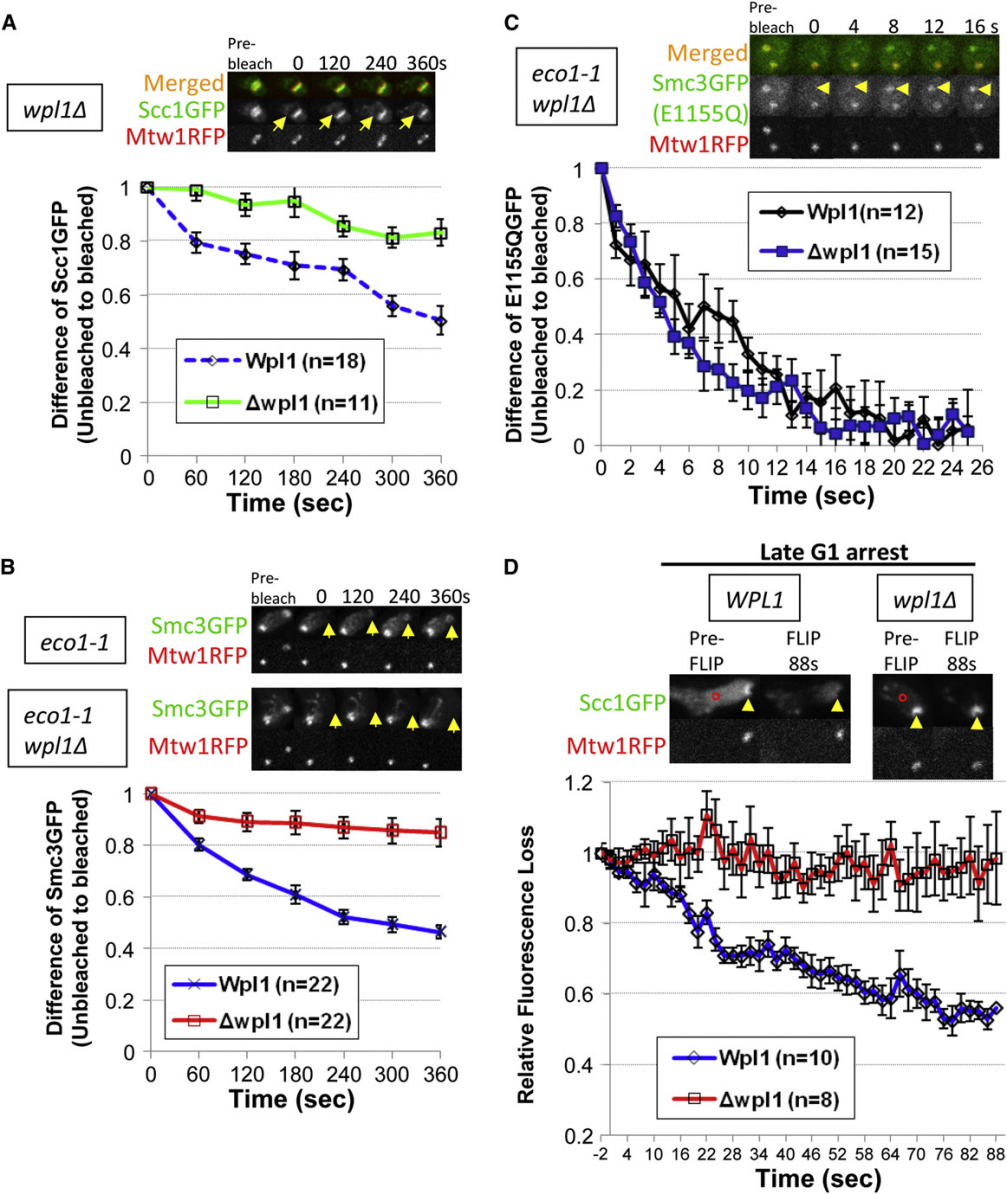
Pericentric Cohesin Turnover Depends on Wapl (A) Dynamics of pericentric Scc1-GFP in *WPL1*^*+*^ (K18246), previously shown in Figure 3C, and *wpl1Δ* (K18754) tetraploids. (B) Dynamics of pericentric Smc3-GFP in *eco1-1* (K18454) and *eco1-1 wpl1Δ* (K18784) diploids. Cells were preincubated at the nonpermissive temperature for 90 min to inactive *eco1-1*. (C) Dynamics of ATP-hydrolysis mutant Smc3E1155Q in *eco1-1* (K19362) and *eco1-1 wpl1Δ* (K19037) diploids. Data are represented as mean difference of normalized fluorescence intensity between bleached and unbleached regions ± SEM. n = the number of cells examined. See also Figure S3 for normalized fluorescence intensity of unbleached and bleached regions and the effect of *wpl1Δ* on the amounts of pericentric cohesin in *eco1-1* mutants, respectively. (D) FLIP analysis of pericentric Scc1-GFP in *WPL1*^*+*^ (K19295) and *wpl1Δ* (K19297) *a/a* diploids. Cells were arrested in late G1 by overexpression of nondegradable sic1(9 m) following α-factor release. A single laser beam (red circle) repeatedly photobleached a point outside pericentric regions (arrows). *a/a* diploids were used to minimize off target photobleaching. FLIP was performed at least 60 min after pheromone release to ensure maximum loading of cohesin onto pericentric chromatin. Data are represented as mean relative fluorescence loss (Scc1GFP/Mtw1RFP) at pericentric regions ± SEM. n = the number of cells examined. See also Figures S4 and S5 for late G1 arrest and normalized fluorescence intensity. Sister-centromere clusters are marked by Mtw1RFP.

**Figure S3:**
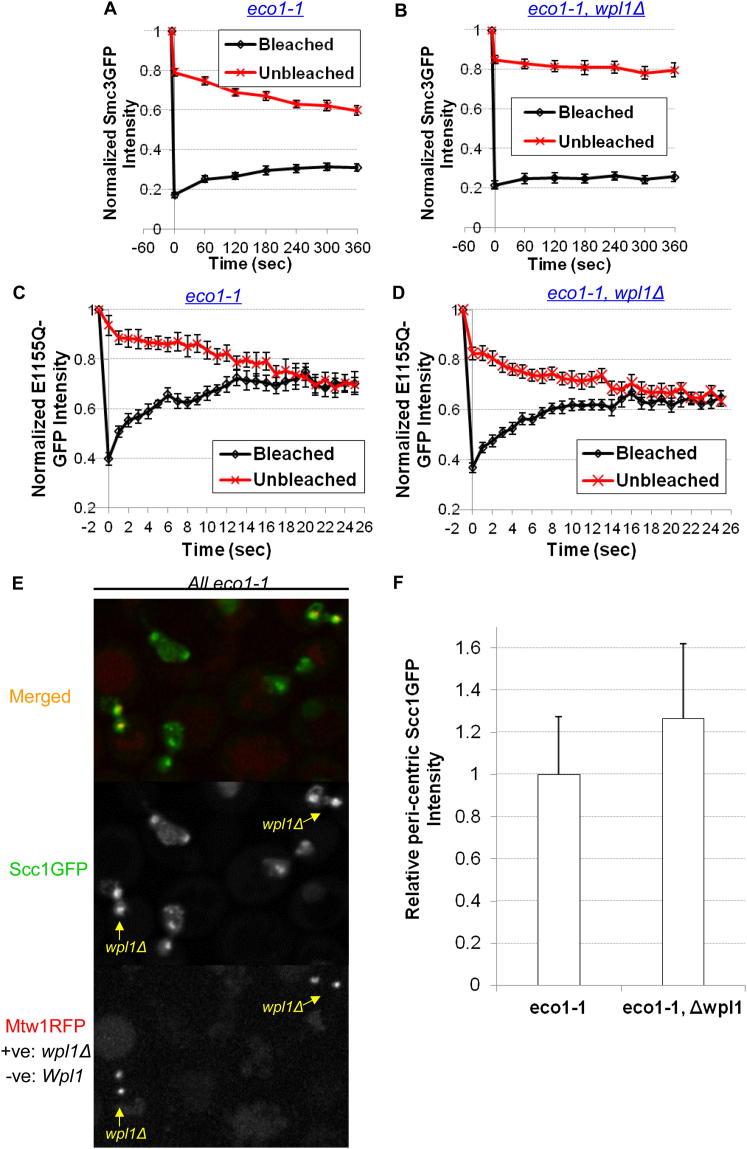
The Effect of *wpl1Δ* on Cohesin Turnover and on the Amounts of Pericentric Cohesin in *eco1-1* Diploids at Restrictive Temperature, Related to Figure 4 (A and B) Normalized Smc3GFP intensity of unbleached and bleached centromere foci in *eco1-1* (A, K18454) and *eco1-1 wpl1Δ* (B, K18784) diploids. (C and D) Normalized Smc3(E1155Q)GFP intensity of unbleached and bleached centromere clusters in *eco1-1* (C, K19362) and *eco1-1 wpl1Δ* (D, K19037). Data are represented as mean normalized fluorescence intensity (to prebleaching regions) ± SEM. n = the number of cells examined. (E) Representative images showing pericentric Scc1GFP in *eco1-1* (K18337, Mtw1RFP: –ve) and *eco1-1 wpl1Δ* (K19255, Mtw1RFP: +ve) diploids. Live-cell imaging was performed on the same slides after cells preincubated at restrictive temperature for 1.5 hr to inactivate *eco1*. (F) Quantitation of the relative pericentric Scc1GFP intensity in (E).

Detection of pericentric cohesin turnover enabled us to test Wapl’s role. *wpl1Δ* largely abolished turnover of Scc1-GFP within the pericentric barrels of tetraploid cells (Figure 4A). It also greatly reduced dissociation in *eco1-1* cells preincubated at the restrictive temperature (Figure 4B; Figures S3A and 3B). Though *wpl1Δ* suppresses *eco1-1* lethality, it does not prevent disjunction of most pericentric Smc3-GFP to spindle poles, which therefore forms two foci of fluorescence. However, due to restoration of some cohesion, these remain connected by fine cohesin-rich threads. Crucially, iFRAP revealed little if any reduction in the fluorescence of Smc3-GFP at unbleached poles in *eco1-1 wpl1Δ* cells (Figure S3B), implying little or no turnover. In contrast to the striking effect on wild-type cohesin dynamics, Wapl had no effect on the rapid turnover at centromeres (Hu et al., 2011) of complexes defective in hydrolysis of ATP bound to Smc3 NBDs (Figure 4C; Figures S3C and 3D), implying that Wapl only promotes turnover of cohesin complexes that have fully completed chromatin loading.

Because about one fifth of pericentric cohesin does not turn over even in *WPL1 eco1-1* cells, the reduced turnover in *wpl1Δ* cells could be caused by selective degradation of the dynamic pool. This cannot be the explanation because loss of Wapl slightly increases not decreases the amount of pericentric cohesin in *eco1-1* cells (Figures S3E and 3F).

**Figure S4:**
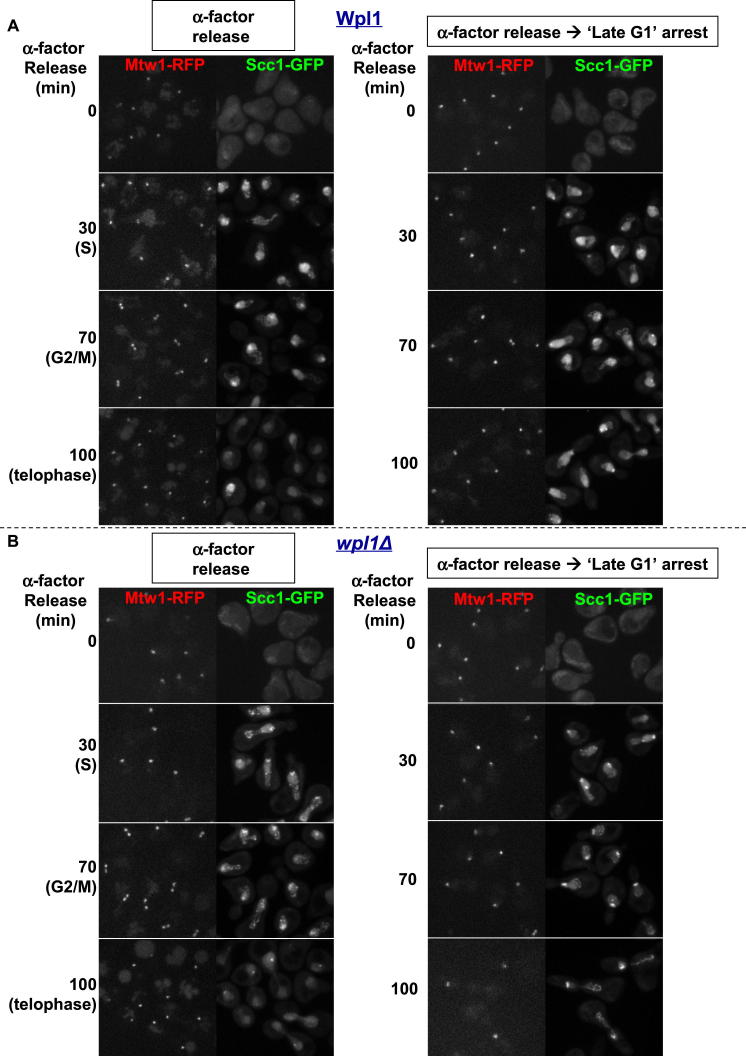
Live-Cell Imaging of the Expression and Localization of Scc1GFP after Pheromone Release to “Late G1” Arrest, Related Figure 4D (A) Wild-type cells (K19134) expressing Scc1GFP and Mtw1RFP were arrested in α-factor for 3.5 hr. *Gal1p-Sic1(9 m)* was overexpressed (right) or not (left) for 2 hr before pheromone release by addition of galactose. Live-cell imaging was performed at indicated time points. (B) Same as (A) except using *wpl1Δ* cells (K19137).

**Figure S5:**
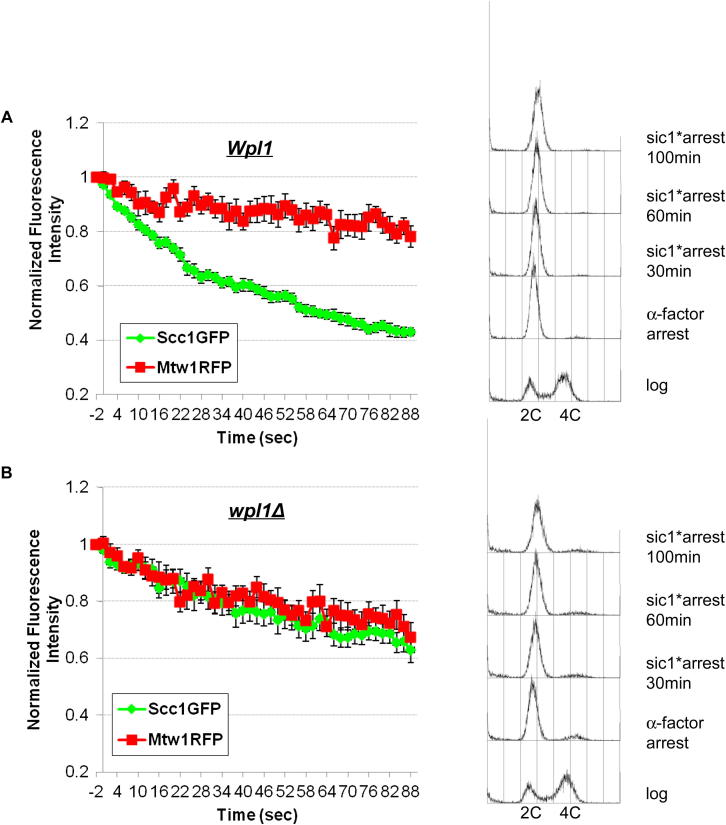
FLIP Analysis of Pericentric Cohesin in “Late G1” Arrested Wild-Type and *wpl1Δ* Diploid Cells, Related to Figure 4D (A and B) FLIP analysis showed the normalized Scc1GFP and Mtw1RFP fluorescence intensity in wild-type (A, K19295) and *wpl1Δ* (B, K19297). Cells were arrested in α-factor for 2.5 hr. *Sic1(9 m)* protein was overexpressed for 1 hr before pheromone release. FLIP was performed after 60 min in “late G1” arrest. FACS profiles were shown on right. Data are represented as mean normalized fluorescence intensity (to prebleaching regions) ± SEM. n = the number of cells examined.

Because *wpl1Δ* rescues cohesion in *eco1-1* cells, it is important to confirm that the reduced cohesin turnover is not an indirect consequence of cohesion establishment. We therefore also measured Wapl’s effect on cohesin dynamics in cells arrested in late G1 when there is also little or no Smc3 acetylation but no cohesion (data not shown). To do this, we expressed a nondegradable version of a Cdk inhibitor, *sic1(9 m)* by using an improved galactose-inducible system (Matsuyama et al., 2011; Nash et al., 2001). Cells released from pheromone induced G1 arrest in the presence of galactose re-enter the cell cycle, form buds, and resynthesize Scc1, but fail to enter S phase due to inhibition of Clb/Cdk1 kinases. Western blotting showed that *wpl1Δ* reduces modestly reaccumulation of Scc1 protein (data not shown), but this has little effect on the amount of Scc1-GFP associated with pericentric regions (Figure S4). We compared its dynamics in the presence and absence of Wapl by measuring the effect of repeatedly photobleaching the opposite half of the nucleus (FLIP). If pericentric Scc1-GFP turns over then it will rapidly enter the half being photobleached, and pericentric fluorescence will decay. Strikingly, this decay was almost entirely eliminated by *wpl1Δ* (Figure 4D; Figure S5), confirming Wapl’s role in releasing unacetylated cohesin from pericentric chromatin.

### Cohesin Turnover Is Reduced by All Antiestablishment Mutations

**Figure 5:**
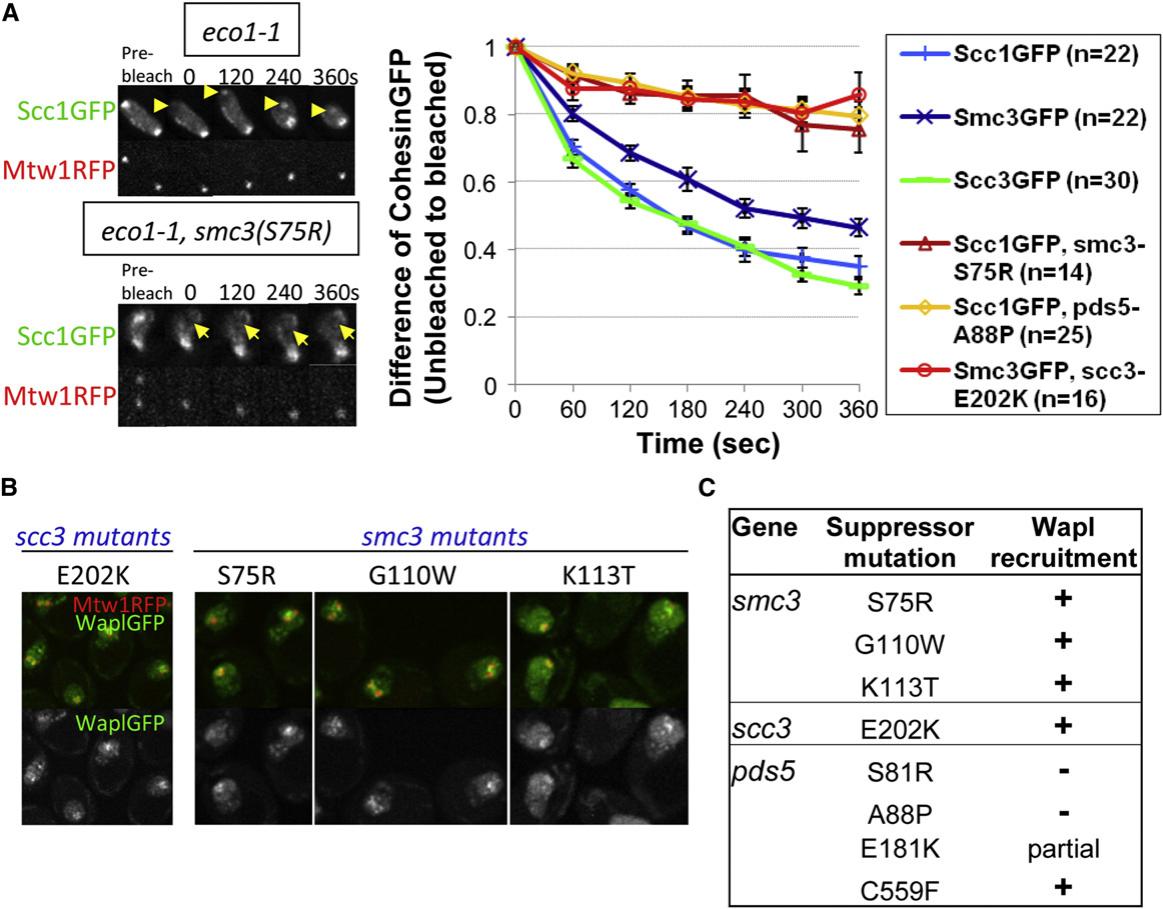
Cohesin Turnover Is Reduced by Antiestablishment Mutations (A) Dynamics of pericentric cohesin in *eco1-1* (K18402, K18454, and K18455) and in *eco1-1 smc3S75R* (K19306), *eco1-1 pds5A88P* (K19066), and *eco1-1 scc3E202K* (K19203) diploids. Cells were preincubated at the restrictive temperature for 90 min before photobleaching. Data are represented as mean difference of normalized fluorescence intensity between bleached and unbleached regions ± SEM. n = the number of cells examined. See also Figure S6 for normalized fluorescence intensity of unbleached and bleached regions. (B) Wapl-GFP localization in “antiestablishment” mutants (K19253, K19254, K19257, and K19252). (C) Summary of pericentric recruitment of Wapl in (B).

**Figure S6:**
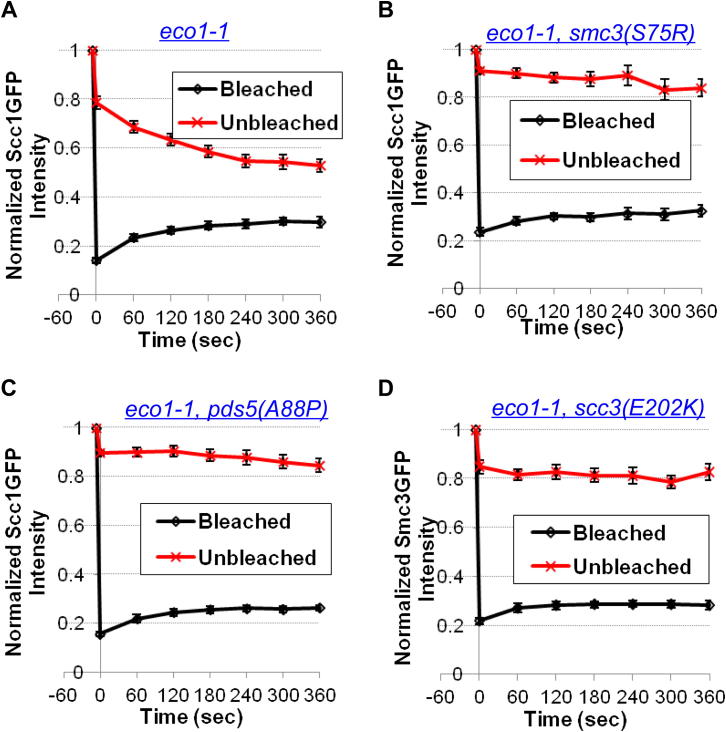
The Effect of Antiestablishment Mutations on Cohesin Turnover in *eco1-1* Diploids at Restrictive Temperature, Related to Figure 5 (A–D) Normalized cohesin fluorescence intensity of unbleached and bleached centromere clusters in *eco1-1* (A, K18402) and in *eco1-1* diploids containing Smc3S75R (B, K19306), Pds5A88P (C, K19066) and Scc3E202K (D, K19203). Data are represented as mean normalized fluorescence intensity (to prebleaching regions) ± SEM. n = the number of cells examined.

The above observations imply that yeast cohesin has a releasing activity despite lacking a prophase pathway. If this activity is synonymous with antiestablishment, then all mutations that suppress *eco1-1* lethality should reduce pericentric cohesin dissociation, even in *WPL1*^*+*^ cells. We therefore measured the effect on turnover in *eco1-1* cells of *smc3(S75R)*, *pds5(A88P)*, and *scc3(E202K)*. Remarkably, all three types of *eco1* suppressor mutation greatly reduced turnover (Figure 5A). Despite a modest recovery of fluorescence within bleached foci, possibly due to diffusion of soluble complexes, there was little or no convergence of levels at bleached and unbleached poles, indicating that cohesin associated with each is stably bound (Figures S6). To address whether the turnover defects of these mutants are caused by a failure to recruit Wapl, we analyzed their effect on Wapl-GFP distribution. As already described, mutations within Pds5’s N-terminal domain, namely S81R and A88P abolished localization to pericentric chromatin, whereas E181K reduced it (Figure 2E). In contrast, Pds5C599K had little or no effect (Figure 2E) nor did Scc3E202K, Smc3S75R, G110W or K113T mutations (Figure 5B and 5C).

### Fusion of Smc3 to α-Kleisin Protects Cohesion from Cohesin’s Releasing Activity

Our experiments imply that cohesin’s releasing activity not only promotes cohesin’s turnover on chromosomes but also destroys cohesion once it has been established. The ring model has a simple explanation for this duality, namely that the activity triggers escape of DNAs from the Smc1/Smc3/α-kleisin ring, presumably by opening one of its three interfaces. If so, preventing the opening of this interface should block release and, in so far as Eco1 hinders release, should bypass the need for Eco1-mediated Smc3 acetylation. To test whether Smc NBD/kleisin interfaces are involved in release, we compared the effect of fusing either Smc3’s C terminus to α-kleisin’s N terminus or α-kleisin’s C terminus to Smc1’s N terminus, which should block putative exit gates at Smc3/kleisin and Smc1/kleisin interfaces, respectively. It is important to note that neither of these two fusions (Gruber et al., 2006) nor the loss of releasing activity is lethal in *S. cerevisiae*.

**Figure 6:**
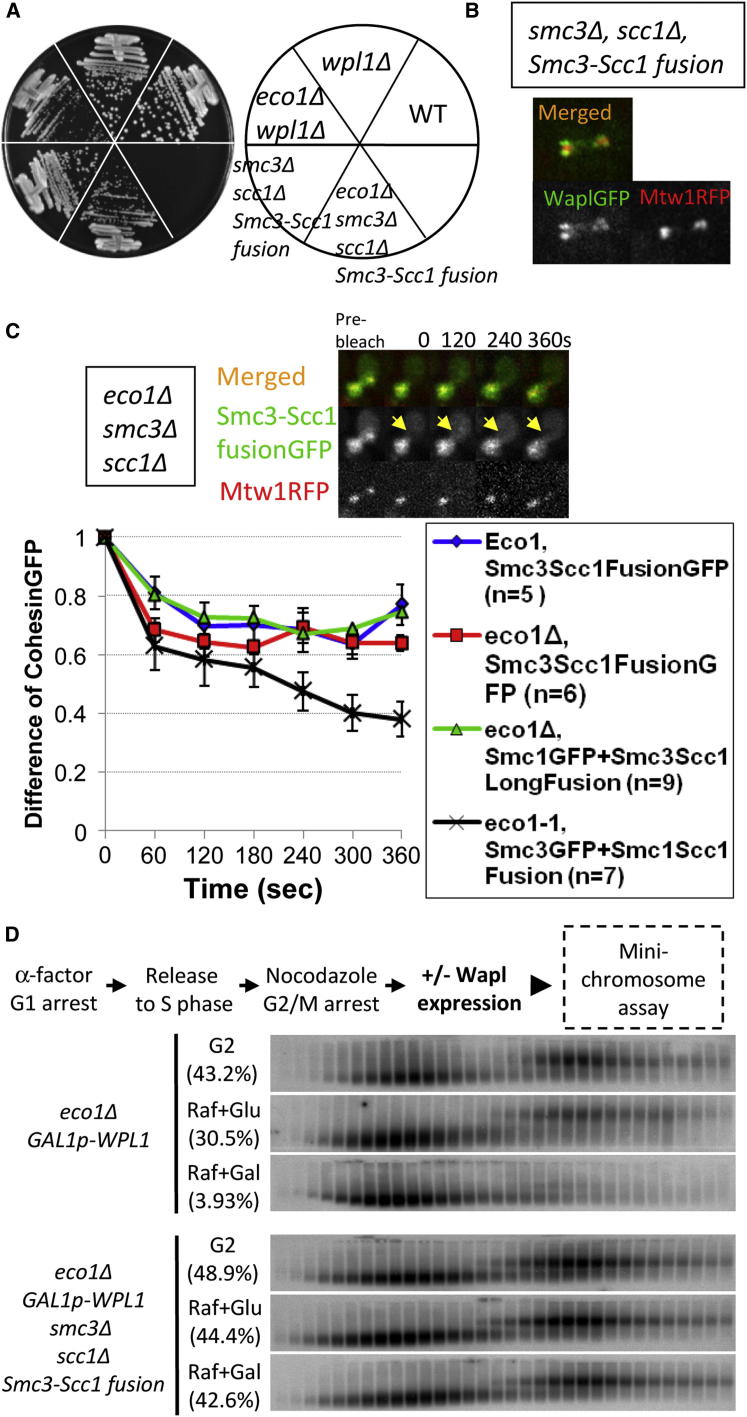
Fusion of Smc3 to α-Kleisin Protects Cohesin from Its Releasing Activity (A) Cells expressing the Smc3-Scc1 fusion protein rescue *eco1Δ* lethality (K699, K18742, K16431, K16292, and K16460). (B) Pericentric localization of Wapl-GFP in live cells expressing Smc3-Scc1 fusion protein (K19495). (C) Fusion of Smc3 to Scc1, but not Scc1 to Smc1, reduces turnover of pericentric cohesin (arrows). Dynamics of pericentric Smc3-Scc1-GFP fusion proteins in wild-type *ECO1* (K19176) or *eco1Δ* (K19377) cells, of Smc1-GFP in *eco1Δ Smc3-Scc1 long-linker fusion* (K19491) cells, and of Smc3-GFP in *eco1-1 Scc1-Smc1 fusion* (K19514) cells. Data are represented as mean difference of normalized fluorescence intensity between unbleached and bleached clusters ± SEM. n = the number of cells examined. Sister-centromere clusters are marked by Mtw1RFP. See also Figure S7 for normalized fluorescence intensity unbleached and bleached regions. (D) Wapl induction during G2/M destroys cohesion in *eco1Δ* cells (K18943) but not in *eco1Δ* cells expressing an Smc3-Scc1 fusion protein (K19129). Minichromosome cohesion assay as described for Figure 1B. Brackets denote percentages of DNAs in dimer fractions.

**Figure S7:**
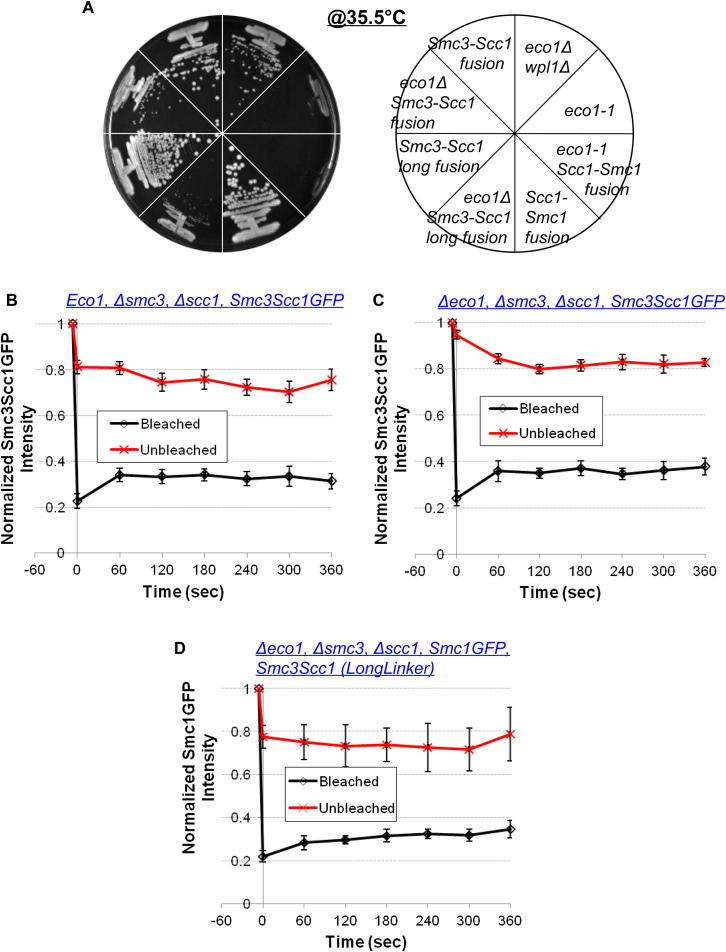
The Effect of Smc-Scc1 Fusion on Viability and Cohesin Dynamics in *ECO1+* and *eco1Δ* Cells, Related to Figure 6C (A) Cells expressing the Smc3-Scc1 fusion protein rescue *eco1Δ* lethality. *eco1-1* (K18565) or *eco1Δ* cells with indicated Smc-Scc1 fusion proteins (K16292, K16460, K19313, K19463, K19221, and K19500) or *wpl1Δ* (K16431) were grown on YEPD plates at restrictive temperature (35.5°C). (B–D) Dynamics of pericentric Smc3-Scc1-GFP fusion proteins in *ECO1+* (B, K19176) or *Δeco1* (C, K19377) cells, and pericentric Smc1-GFP in *Δeco1 Smc3-Scc1 long-linker fusion* (D, K19491) cells. Data are represented as mean normalized fluorescence intensity (to prebleaching regions) ± SEM. n = the number of cells examined.

Remarkably, tetrad dissection revealed that fusion of α-kleisin to Smc3 but not to Smc1 suppressed lethality due to *eco1Δ* (Figure 6A) or *eco1-1* (Figure S7A). The same selective suppression by Smc3/α-kleisin but not Smc1/α-kleisin fusions was observed when the linkers connecting Smc NBDs and α-kleisin (Scc1) were increased from 44 to 71 residues (Figure 6A). Because suppression by the Smc3-Scc1 fusion protein is abolished by TEV-induced cleavage of its linker (data not shown), it cannot be attributed to an adventitious juxtaposition of novel amino acids at the C and N termini of Smc3 and Scc1, respectively. Importantly, suppression occurred despite recruitment of Wapl to pericentric cohesin (Figure 6B).

If suppression due to fusion of Smc3 to α-kleisin works by preventing exit of DNAs from cohesin rings, then the fusion should reduce cohesin turnover. iFRAP of cells expressing Smc3-Scc1-GFP fusion proteins or Smc1-GFP together with Smc3-Scc1 fusion proteins with longer linker sequences revealed that the majority (approximately 70%) was stable, even in *eco1Δ* cells (Figure 6C; Figure S7B–S7D). In contrast, fusion of Scc1 to Smc1 had no such effect on turnover of Smc3GFP in *eco1-1* strains preincubated at the restrictive temperature (Figure 6C). Crucially, the stable fraction in cells expressing Smc3-Scc1 fusion proteins was substantially greater than that in cells expressing separate Smc3 and Scc1 proteins, implying that the fusion does indeed reduce turnover. The experiment revealed rapid recovery of about 30% of fluorescence associated with Smc3-Scc1-GFP or Smc1GFP in the vicinity of centromeres. Because the t_1/2_ of this fraction (<30 s) is shorter than that of the mobile fraction of wild-type cohesin (t_1/2_ ∼120 s), it is possible that the recovery observed is due to diffusion of proteins that had never in fact been loaded onto chromatin. For reasons that are not understood, the Smc3-α-kleisin fusion is partially dysfunctional (Gruber et al., 2006), and this appears to reduce the fraction bound to chromatin. Crucially, we never observed reappearance of the bleached pericentric foci, which would have more clearly indicated mobility of proteins associated with centromeres.

If, as the above experiments suggest, fusion of Smc3 to α-kleisin mimics Smc3 NBD acetylation, then sister chromatid minichromosome cohesion generated in the absence of Eco1 and Wapl should be resistant to Wapl reactivation. To test this, Wapl was induced in *eco1Δ GAL-Wpl1* cells that had been allowed undergo S phase in noninducing raffinose medium and then arrested in G2/M. Induction of Wapl in this manner greatly reduced the fraction of dimeric minichromosomes in cells expressing separate Smc3 and α-kleisin proteins (Figure 6D, upper) but had little or no effect in cells expressing an Smc3-α-kleisin fusion protein (Figure 6D, lower). The simplest explanation for this result is that, in the absence of Eco1, cohesin’s releasing activity destroys cohesion by disconnecting the Smc3/α-kleisin interface, permitting DNAs to escape from cohesin’s embrace. Eco1’s function is to inhibit this process by acetylating K112 and K113 within Smc3’s NBD, a process that can be substituted by fusion of Smc3’s NBD to α-kleisin.

## Discussion

Ever since the discovery that the Eco1 acetyltransferase is essential for creating sister chromatid cohesion but not for loading cohesin on to chromosomes (Tóth et al., 1999), it has been clear that this enzyme (Ivanov et al., 2002) is central to understanding how cohesin holds sister DNAs together. The enzyme exerts its effect by acetylating Smc3’s NBD at K112 and K113. Remarkably, this normally essential modification can be mimicked and the lethality of *eco1* mutants suppressed either by null alleles of Wapl or by highly specific missense mutations within Smc3, Pds5, and Scc3. This has led to the notion that Eco1 counteracts an activity intrinsic to cohesin that hinders its ability to build stable cohesion. A critical issue is whether this “antiestablishment” activity prevents creation of cohesive structures in the first place or merely destroys them after their creation during S phase (Rowland et al., 2009). Our finding that Wapl is able to destroy cohesion long after replication is complete in cells lacking Eco1 is consistent with the latter hypothesis.

How then does antiestablishment destroy cohesion? The answer is suggested by our finding that a large fraction of unacetylated cohesin complexes associated with pericentric chromatin turns over and that this process is greatly reduced by mutations that bypass the need for Eco1, implying that antiestablishment is synonymous with a feature of cohesin that enables it to disengage from chromatin in the absence of α-kleisin cleavage (Gerlich et al., 2006). In other words, it is cohesin’s “releasing activity” that destroys cohesion built in the absence of Smc3 acetylation. In mammalian cells, Wapl depletion not only reduces cohesin’s turnover on chromosomes but also increases the fraction of cohesin associated with chromatin, especially as cells enter mitosis (Kueng et al., 2006). Surprisingly, less not more cohesin is found associated with yeast chromosomes in *wpl1Δ* cells. An explanation for this phenomenon is that Scc1 protein levels are reduced about two-fold in *wpl1Δ* cells, possibly due to lower rates of synthesis in late G1. The cause of this phenomenon is currently under investigation.

If releasing activity is an inherent aspect of cohesin complexes, then it is equally vital that cells possess a mechanism to neutralize it in a subset of cohesin complexes that entrap sister DNAs during replication. We suggest that this is the function of acetylation of Smc3 NBDs by Eco1, a model similar to that proposed for animal cells where acetylation has been proposed to block Wapl activity by recruiting sororin (Lafont et al., 2010; Nishiyama et al., 2010). According to this notion, Smc3 acetylation is required to keep releasing activity continuously in check and is therefore responsible for maintaining cohesion after S phase. Our finding that partial deacetylation of Smc3 due to Hos1 overexpression is accompanied by reduced sister chromatid cohesion (Beckouët et al., 2010) is consistent with this. We currently have no explanation for the finding that about 20% of pericentric cohesin fails to turn over even in the absence of Eco1. If inactivating cohesin’s releasing activity is Eco1’s sole function, then why does this stable pool of cohesin complexes not support viable chromosome segregation?

If as envisaged by the ring model, cohesin’s stable or even semistable chromosomal association is mediated by entrapment of chromatin fibers, then release must involve their escape. The ring must have a DNA exit gate. Our finding that cohesin containing an Smc3-kleisin fusion protein fails to turn over once it has associated with chromatin and moreover creates cohesion without Eco1 suggests that the exit gate is situated at the Smc3/kleisin interface. A corollary is that the primary function of Smc3’s NBD acetylation by Eco1 is to block dissociation of cohesin’s Smc3/kleisin interface, which ensures that DNAs remain entrapped by cohesin’s tripartite ring. This conclusion implies that DNA exit catalyzed by cohesin’s releasing activity is not simply a reversal of entry (Nishiyama et al., 2010). The ring must have separate DNA entry and exit gates.

Because it is the only component of cohesin’s releasing activity that does not also have roles in establishing or maintaining cohesion, Wapl may be rather directly involved disengaging α-kleisin’s N-terminal domain from Smc3 NBDs. How might it perform this task? One possibility is that, with help from Pds5 and Scc3, Wapl binds to the Smc3 NBD in a manner that precludes α-kleisin binding. Thus, Wapl and α-kleisin might compete for binding to the same site on Smc3. Elucidating how Smc3’s NBD binds to α-kleisin will be vital to understanding this process. Crucially, we suggest that K112 and K113 within Smc3’s NBD have a key destabilizing influence on its interaction with α-kleisin’s N-terminal domain and that this effect (whether direct or indirect) is neutralized by acetylation. Unlike α-kleisin, which will be bound to the complex via its C-terminal domain throughout the disengagement cycle, Wapl is never stably associated with cohesin and its displacement of α-kleisin from Smc3 can therefore only be a temporary event.

It is important to stress that releasing activity is not intrinsic to the Smc3/kleisin interface or even to the influence of Wapl. It also needs residues within Pds5 and Scc3 that are not required for Smc3’s association with α-kleisin or for recruiting Wapl. How Scc3 and Pds5 regulate dissociation allosterically and whether the process also involves binding and/or hydrolysis of ATP to Smc NBDs are crucial issues for future research. Another is whether the rapid turnover of Wapl within cohesin, which is far more rapid than that of its partner Pds5, is an intrinsic aspect of cohesin’s releasing activity.

**Figure 7:**
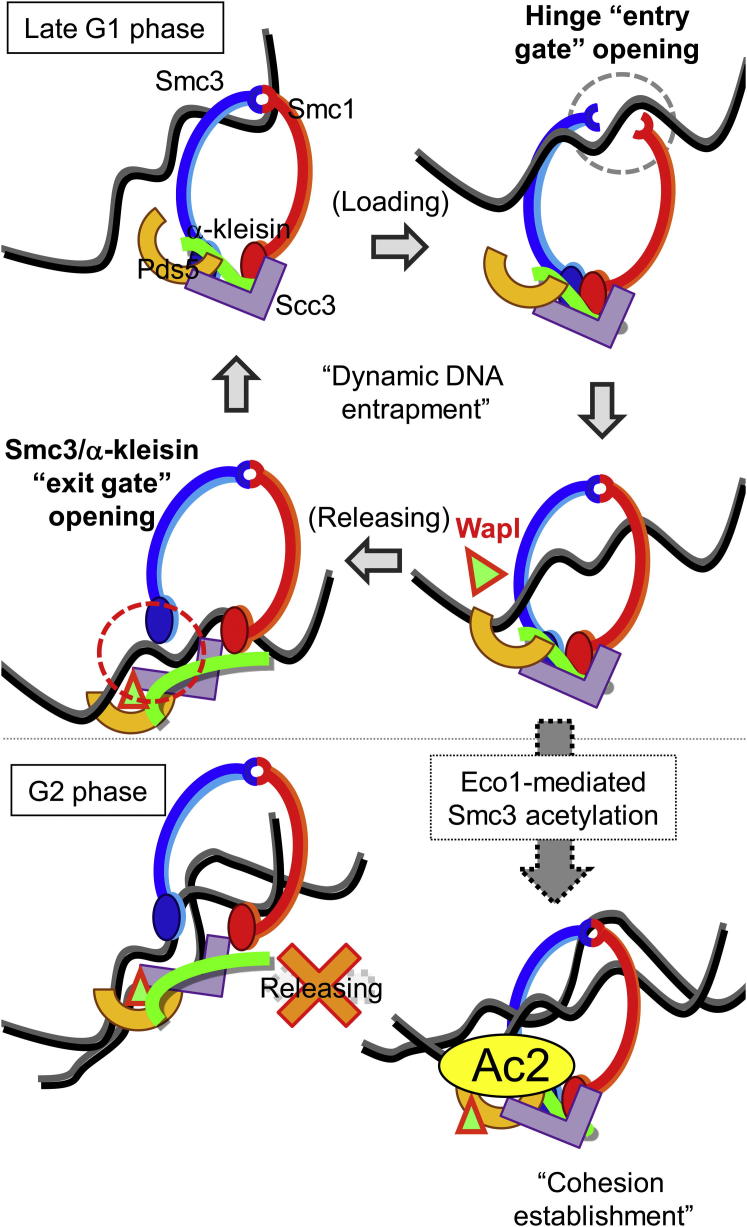
A Model: Acetylation of Smc3 NBDs by Eco1 Prevents Transient Dissociation of the Smc3/Kleisin Interface and Thereby Blocks Escape of DNAs Scc1 synthesis in late G1 leads to cohesin’s loading onto chromatin due to transient opening of its hinge domains by Kollerin (Scc2/4). Wapl acts with Pds5, Scc3, and Smc3 NBDs to release DNA from cohesin rings by opening the Smc3/α-kleisin interface. Free cohesin molecules are reloaded onto DNA. During S phase, acetylation on Smc3 (K112 and K113) by Eco1 prevents dissociation of the Smc3/α-kleisin interface and thereby maintains sister DNAs inside cohesin rings.

According to our version of the ring model (Figure 7B), acetylation should be viewed as a key that locks cohesin rings shut once DNAs have been trapped inside. This process is coupled to DNA replication (Rolef Ben-Shahar et al., 2008) and in yeast is not reversed until cohesin rings are cleaved by separase (Beckouët et al., 2010). The locked acetylated state may need to be extraordinarily robust, especially in cells such as oocytes where the cohesion holding bivalent chromosomes together may need to last for several weeks if not decades (Tachibana-Konwalski et al., 2010). In mammalian cells, acetylation is insufficient to neutralize cohesin’s releasing activity. The modification promotes recruitment of sororin, which is thought to displace Wapl from its binding site on Pds5 (Lafont et al., 2010; Nishiyama et al., 2010). If sororin does not exist in yeast, which is not known for sure, then acetylation must alter some other aspect of cohesin. Our finding that turnover of a fraction of Pds5 molecules within pericentric chromatin is greatly reduced by Eco1 activity suggests that Smc3 acetylation alters the way Pds5 interacts with cohesin, a change that might have a role in neutralizing releasing activity.

There are good reasons to believe that the mechanism by which cohesin dissociates from yeast chromosomes via opening the Smc3/α-kleisin interface will apply to the “prophase pathway” that removes most cohesin from chromosome arms as animal cells enter mitosis. Because Wapl in mammals is required for cohesin’s turnover on chromatin during interphase as well as during prophase (Kueng et al., 2006), it is likely that cohesin’s depletion from chromosome arms during prophase is triggered by hyperactivation of the same releasing activity that merely induces turnover during interphase. Interestingly, the prophase pathway is also dependent on phosphorylation of SA (Scc3) proteins (Hauf et al., 2005), emphasizing that this subunit is intimately involved in releasing activity in animal cells as well as yeast.

The finding that yeast cells lacking releasing activity are viable raises the question as to why it is such a conserved feature of eukaryotic cohesin complexes. If it did not exist, there would be less or possibly no necessity for Eco1. Indeed, some eukaryotic organisms appear to lack both proteins (Nasmyth and Schleiffer, 2004). It may be relevant in this regard that cohesin has functions besides mediating sister chromatid cohesion (Kagey et al., 2010; Parelho et al., 2008; Wendt et al., 2008) and is important in nonproliferating as well as proliferating cells (Pauli et al., 2008; Seitan et al., 2011). It is thought that cohesin has important roles in regulating transcription, presumably through modulating the topology of interphase chromatin. Due to the dynamic nature of the transcription process, it is inconceivable that such functions could be mediated by cohesin complexes lacking a capacity for turning over. We suggest that a dynamic entrapment of chromatins is important for achieving the appropriate distribution of cohesin complexes along the genome; remodeling intrachromatid loops; removing what could be topological barriers under certain conditions during transcription, repair, or replication; and dissolving inappropriate connections between nonsister chromatids. This is in addition to the value of protecting a large fraction of the cohesin pool from separase as a consequence of its prior dissociation from chromosomes during prophase (Kucej and Zou, 2010). Given that releasing activity destroys a state catalyzed by kollerin, it is possible that defects caused by haploinsufficiency of kollerin’s Scc2/Nipbl subunit (Pehlivan et al., 2012) that are characteristic of Cornelia de Lange syndrome might be caused at least in part by releasing activity. If so, partial inhibition could conceivably alleviate any nondevelopmental symptoms.

Our finding that cohesin’s dissociation from chromatin involves opening the Smc3/kleisin interface is an important endorsement of the ring model. It also provides a theoretical framework for exploring how release is regulated by acetylation during S phase and by phosphorylation during mitosis. It may also have important implications for other eukaryotic Smc/kleisin complexes. Because the N-terminal domains of kleisins are highly conserved, it is conceivable that their regulated association with and dissociation from their cognate Smc NBDs will prove to be a conserved feature of these chromosomal machines. In which case, the topological principles according to which cohesin functions may apply also to condensin and Smc5/6 complexes.

## Experimental Procedures

Additional details were described in the Extended Experimental Procedures.

### Yeast Culture and Cohesion Assay

All strains are derivatives of W303 (K699). The detailed genotypes were in the Supplementary Information (see Table S1). Cells were cultured in YEP medium with 2% glucose unless otherwise stated. Cohesion assay was performed as described in Farcas et al. (2011) with a 7.5 kb circular minichromosome. Briefly, the cells were lysed, and extracts were fractionated by sucrose gradient centrifugation. Minichromosome DNA were separated by gel electrophoresis and detected by Southern blotting.

### Live-Cell Imaging, Photobleaching, and Image Analysis

Exponentially growing cells were placed on agarose pads, and fresh samples were prepared every 15 min for all microscopy experiments. Live-cell imaging was performed in a spinning disk confocal system (PerkinElmer UltraVIEW) with an EMCCD (Hamamatsu) mounted on an Olympus IX8 microscope with Olympus 60× 1.4 N.A. and 100× 1.35 N.A. objectives. Image acquisition and quantitation was done by using Volocity software. iFRAP was carried out with a 488 nm laser beam, 100% power, 15–30 ms. Fluorescence intensity measurement was performed by using ImageJ. All signals were subjected to background correction. Fluorescence intensity of unbleached and bleached areas was normalized to that of initial prebleaching images (See Figures S2, S3, S6, and S7).

$$\text{Difference}={\text{NI}}_{\text{unbleach}}-{\text{NI}}_{\text{bleach}},\;\text{where}\;\text{NI}\;\text{represents}\;\text{normalized}\;\text{intensity}$$

The difference at time = 0 (bleaching) was set as 1. Data were represented as mean difference ± SEM. Mobile fraction was estimated from the proportion of the mean difference declined at equilibrium or at the last time point. Half-life (t_1/2_) was calculated as the time required for 50% decay of the mean fluorescence difference in the mobile fraction.

FLIP was performed by repeatedly pulse bleaching with 10% of 488 nm laser power, 30 ms. Fluorescence intensity was measured by using Volocity. Pericentric GFP and RFP fluorescence was background corrected and normalized to that of initial pre-FLIP images (See Figure S5). Relative fluorescence loss was calculated as a ratio of normalized intensity of GFP to that of RFP.

Extended Experimental Procedures **Live-Cell Imaging and Fluorescence Comparison Analysis**

All strains used for live-cell imaging are diploid. Exponentially growing cells were placed on 2.5% agarose pads made of synthetic complete medium plus glucose or galactose. Live-cell imaging was performed under a spinning disk confocal system (PerkinElmer UltraVIEW) with an EMCCD camera (Hamamatsu) mounted on an Olympus IX8 microscope with Olympus 60× 1.4 N.A. and 100× 1.35 N.A. objectives. Image acquisition was done at 25°C. For fluorescence comparison analysis of strains with different cohesin subunits tagged with GFP, cells were grown to mid-log phase with OD = 0.3, and mixed together and placed on the same agarose pads. Seventeen Z stacking images with 0.2 μm intervals were acquired and quantitation was done by using Volocity software. All signals were subjected to background correction. Fresh samples were prepared every 15 min.

**Inverse Fluorescence Recovery after Photobleaching Analysis**

All strains used for photobleaching are diploids and tetraploids. Cells were grown in YEP-2% glucose at 25°C. For *eco1-1* strains, a preincubation at restrictive temperature 25°C (35.5°C) for 1.5 hr was required before subjecting to photobleaching. Cells were placed on 2.5% agarose pads made of synthetic complete medium plus glucose. Photobleaching was carried out under 100× objective with a 488 nm laser beam, 100% power, 30 ms (or 15 ms for Wapl and Smc3 ATP-hydrolysis mutant E1155Q proteins) at 25°C. A region with size equal to a single laser beam (<700 nm diameter) or slightly larger was selected. Single-stacking images were acquired for Wapl-GFP and Smc3 (E1155Q)-GFP, and 5 Z stacking images with 0.4 μm intervals were acquired for all other cohesion subunits by using Volocity. Intensity measurement was performed by using ImageJ. Mean GFP fluorescence intensity of unbleached and bleached areas was background corrected, and normalized to that of prebleaching images. The difference between normalized intensity of unbleached and bleached areas at prebleaching is 0 and postbleaching (time = 0) is set as 1. Fresh samples were prepared every 15 min.

**Fluorescence Loss in Photobleaching**

All strains used for FLIP are *a/a* diploids. Cells were first arrested in YEP-2% raffinose with α-factor for 2.5 hr at 25°C. Overexpression of *GAL1p-sic1(9 m)* was done 1 hr before pheromone release by addition of 2% galactose. Cells were released in YEP with 2% galactose for at least 60 min before FLIP. A point with size equal to a single laser beam (<700 nm diameter) and approximately 3.5 μm away from pericentric region was photobleached. FLIP was performed under 100× objective by repeatedly pulse bleaching with a single 488 nm laser beam (10% power, 30 ms). Single-stack images were acquired during FLIP. Intensity measurement was done by using Volocity software. Pericentric GFP and RFP fluorescence was background corrected, and normalized to that of pre-FLIP images. Relative fluorescence loss was calculated as a ratio between normalized GFP to normalized RFP intensity. Fresh samples were prepared every 15 min.

## Supplemental Information

Document S1. Table S1

Document S2. Article plus Supplemental Information
